# The quest for universal access to effective malaria treatment: how can the AMFm contribute?

**DOI:** 10.1186/1475-2875-9-274

**Published:** 2010-10-08

**Authors:** Lloyd Matowe, Olusoji Adeyi

**Affiliations:** 1 The Global fund to fight AIDS, Tuberculosis and Malaria, AMFm Unit, Chemin de Blandonnet 8, 1214 Vernier, Geneva, Switzerland

## Abstract

Access to quality assured artemisinin-based combination therapy (ACT) has remained very low in most malaria endemic countries. A number of reasons, including unaffordable prices, have contributed to the low accessibility to these life-saving medicines. The Affordable Medicines Facility-Malaria (AMFm) is a mechanism to increase access to quality assured ACT. The AMFm will use price signals and a combination of public and private sector channels to achieve multiple public health objectives: replacing older and increasingly ineffective anti-malarial medicines, such as chloroquine and sulphadoxine-pyrimethamine with ACT, displacing oral artemisinin monotherapies from the market, and prolonging the lifespan of ACT by reducing the likelihood of resistance to artemisinin.

Access to medicines frameworks paint a broad picture of dimensions of access to medicines and juxtapose components that enhance or hinder access to medicines. Access requires various activities--funding, institutions, interventions, and thinking--from public and private actors at global, national, and local levels. This paper examines, within access to medicines frameworks, the role of the AMFm across and within each dimension and discusses how the AMFm can help to solve access bottlenecks.

## Background

Universal access to effective malaria treatment is among the United Nations' Millennium Development Goals [1]. This also is among the goals of the Roll Back Malaria Partnership [2]. In spite of high-level commitments, political will and substantial increases in financing, the attainment of this goal has remained elusive in most malaria-endemic countries [3], especially in relation to artemisinin-based combination therapy (ACT), the treatment recommended as first-line by the World Health Organization (WHO) for uncomplicated malaria caused by *Plasmodium falciparum *[4].

In the World Malaria Report 2009 [3], WHO reported that access to ACT was generally poor in African countries, with less than 15 percent of children under five years of age receiving an ACT when they had fever in 11 of 13 African countries for which survey data were available. These findings are consistent with results of more recent multi-country surveys[5], which included findings for the Democratic Republic of Congo and Nigeria, the largest countries with regard to malaria burden and which together account for approximately 36 percent of all estimated malaria cases in the WHO Africa Region [3].

The Affordable Medicines Facility-malaria (AMFm) is a new financing mechanism to expand access to effective malaria treatment [6-8]. A response to the dual challenge of poor access to quality-assured anti-malarial medicines and threats of parasite resistance to treatment [6], the AMFm combines price negotiations with a factory-gate buyer subsidy to reduce the price of ACT. The AMFm will use price signals and a combination of public and private sector channels to achieve multiple public health objectives. These objectives include replacing older and increasingly ineffective anti-malarial medicines, such as chloroquine and sulphadoxine-pyrimethamine with ACT, displacing oral artemisinin monotherapies from the market, and prolonging the lifespan of ACT by reducing the likelihood of resistance to artemisinin [4].

The AMFm is hosted by the Global Fund to Fight AIDS, Tuberculosis and Malaria. The pilot phase of the programme, which includes Cambodia, Ghana, Kenya, Madagascar, Niger, Nigeria, the United Republic of Tanzania and Uganda, is scheduled to last from 2010 to 2012. The AMFm is funded from multiple sources including a co-payment fund of US$216 million, financed by the Bill and Melinda Gates Foundation, the UK Government, and UNITAID. In addition, the Global Fund provides US$127 million to fund supporting interventions at the country level. Using established access to medicines frameworks [9-11], this paper examines how the AMFm can improve access to effective malaria treatment in malaria endemic countries.

## Access to medicines

In May 2008, WHO adopted the global strategy and plan of action on public health, innovation and intellectual property [12]. This strategy includes a focus on improving the delivery of and access to medicines by effectively implementing successful interventions.

Often, interventions to improve access to medicines focus on the ability to acquire and to utilize medicines alone. Yet access to prompt and effective treatment is broader than the ability to acquire medicines or to utilize services. Access to medicines frameworks paint a broad picture of dimensions of access to medicines and juxtapose components that enhance or hinder access to medicines. Most access to medicines frameworks break down access into four main domains; geographical accessibility, availability, affordability and acceptability [9,10]. A more analytical framework by Frost and Reich [11] adds an organizational dimension, architecture, to a supply component. The architecture is a dimension that allows for coordination of the other access components. This paper adopts Frost and Reich's definition of access as "people's ability to obtain and appropriately use good quality health technologies when they are needed" [11]. The Frost and Reich framework adds further value to access frameworks by mapping the specific activities required to enhance access from the global level down to the user level.

Access requires various activities--funding, institutions, interventions, and analyses--from public and private actors at global, national, and local levels. Within access to medicines frameworks this paper examines the role of the AMFm across and within each access dimension (Table 1). Furthermore, the paper analyses and discusses how the AMFm can help to resolve access bottlenecks within each dimension. A major limitation of this analysis is that the AMFm is primarily an architecture for financing commodities and as such does not satisfy all the dimensions of access to medicines. The conclusions, therefore, should be considered in this context.

**Table 1:** Dimensions of access

Dimension	Definition	Attributes
Architecture	Organizational structures and relationships established with the purpose of coordinating and steering access related activities.	• Availability • Affordability • Geographical accessibility
Geographic accessibility	Defined by the relationship between the location of the product or service and the location of the eventual user of the product or service	• User's location • Location of drug outlets • Infrastructural functionality e.g. roads networks.

Physical availability	Defined by the relationship between the type and quantity of product or service needed, and the type and quantity of product or service provided	• Medicines supply • Medicines demand • Supply chain efficiency

Affordability	Defined by the relationship between prices of the products or services and the user's ability to pay for them	• Price of medicines • Price of services • User's income

**Acceptability**	Defined by the relationship between the user's attitudes and expectations about the products and services and the characteristics of products and services	• Quality of products • Quality of services • User's beliefs and attitudes

## Architecture (Table 1)

Providing access requires work by many different individuals and organizations. Architecture refers to the design and structure of a network of executors and activities required to steer and connect the other access streams (availability, affordability, and acceptability). The AMFm is modeled to enhance access to inexpensive, effective and quality-assured ACT. The model involves the Global Fund, as host and manager of the AMFm, negotiating with drug manufacturers to reduce the price of ACT. In addition to negotiating price reductions, the Global Fund pays a proportion of this reduced price (a 'buyer co-payment') directly to eligible manufacturers to further lower the cost to eligible first-line buyers. First-line buyers then pay the remainder of the sales price. First-line buyers are encouraged to pass on the highest possible proportion of this price benefit so that clients are able to purchase ACT across the public, private not-for-profit and for-profit sectors at a price competitive with that of less-effective anti-malaria drugs, such as chloroquine and sulphadoxine-pyrimethamine. The AMFm has the potential to reduce the cost of ACT to US$0.50 for most patients who pay for treatment. While ACT prices are expected to trend sharply downwards from current levels of up to US$6-10 per treatment, variations are expected within and across countries.

## Geographical accessibility

Geographic accessibility is a component of access that refers to the supply of services location in relation to user location. With regard to ACT, this domain refers to the ease of accessing ACT in relation to the distance to be travelled, the mode of transport for the user and the time it takes to reach the service delivery point. In most developing countries, the distance from health facilities is a critical factor influencing the use of formal healthcare [13,14]. In a study in Kenya, Noor *et al *noted a reduction in the number of patients using formal healthcare services as the distance from health facilities increased [14], while a different study, also in Kenya, reported that a third of patients who self-treated said that they would have sought treatment from a health facility if such a facility were near [15].

Related to distance is the question of how remote a community is geographically. A number of studies in malaria endemic areas have shown that most of the approximately 900,000 annual malaria deaths occur in young children in remote rural areas, which are often hard to reach [16-18]. In many parts of Africa, the distribution of public healthcare facilities is uneven, with the most remote parts, which often include the poorest populations, being underserved [16].

## Availability

Availability is defined as "obtainable or accessible and ready for use" [19]. With regard to medicines, availability refers to the physical presence of the medicine at the service delivery point or the extent to which the medicine can be obtained. Even when ACT is provided free-of-charge in public, non-governmental and faith-based healthcare facilities, availability in peripheral public health facilities remains a challenge. In most instances, ACT movement from the central medical stores to the periphery is often irregular and inconsistent. In 2008, a study in Kenya [20] showed that a quarter of public health facilities had none of the nationally-recommended ACT treatment in stock and three-quarters lacked the full range of weight-specific packs required.

The causes of stock-outs vary, but often reflect weak drug management systems in disease-endemic countries. Poorly resourced supply chains, weak stock management practices, and inadequate lead-time planning can threaten the regular availability of drugs in the public health system. In addition, unpredictable flow of funds in many countries, combined with inadequate distribution from central warehouses to peripheral points of care, undermine the ability to ensure that ACT and other essential drugs are always available at the last mile.

## Affordability

Affordability refers to the price of commodities or services in relation to the client's ability to pay. In developing countries, most people who need medicines have to pay for them out of their own pockets. Where the cost of drugs is covered by formal health services, spending on medicines is a major part of the total healthcare budget. Unaffordable prices have been reported as the major barrier to accessing ACT in malaria-endemic countries [21,22]. Other things being equal, the retail prices of medicines are determined by interplay of demand and supply. As such, the AMFm aims to enhance access to ACT by working through market forces to determine the final sales price of quality-assured ACT on the market. It also includes supporting interventions, such as public information campaigns to mitigate the effects of market failures, such as lack of information about ACT [8].

## Acceptability

Acceptability refers to the characteristics of products and services in relation to user attitudes, perceptions or expectations of products and services. Unlike the other domains, acceptability is a qualitative domain. A variety of interrelated socio-economic factors affect product acceptability and hence prompt and effective access to ACT and related medicines [23-26].

Acceptability is often affected by the community's perceptions of the quality of care provided at service delivery points, the community's trust in the healthcare delivery system and their perception of the effectiveness of specific interventions, including medicines. Related to community perceptions of the quality of care are the community's perceptions of the cause of illness. In some societies traditional beliefs can hinder prompt access to effective treatment. For example, whereas convulsions may be a sign of severe malaria, in some parts of Africa they are associated with the supernatural [27-29], which requires the use of traditional healers for its management.

Communities' perceptions of the effectiveness and detrimental effects of different types of treatment may hinder prompt access to the most effective treatment. In some communities healthcare workers may continue to prescribe non-recommended medicines because patients demand it [30]. The above are challenges that cannot be addressed by price reduction alone. Continuous education about the safety and effectiveness of ACT should be scaled-up together with the scale-up of ACT. The AMFm will work with countries and technical partners to scale-up social marketing interventions in malaria endemic countries.

## Enhancing access to quality-assured ACT

Even though ACT may be provided free-of-charge in public and private-not-for profit facilities, the majority of the populations in malaria endemic countries access these life-saving medicines through the private sector, where currently quality-assured ACT is seldom stocked. Large public health facilities, even when they are easily accessible geographically, tend to be overcrowded. Therefore, in addition to the direct costs of services and medicines, clients incur indirect costs in the form of travel and waiting time when they have to visit distant and crowded health facilities. On the other hand, the majority of the population is within reach of a drug shop [31-33]. The World Health Organization approximates the total population that access healthcare services through the private sector to be around 50% in the WHO African and Western Pacific regions and up to 78% in the Southeast Asian region [34]. In Cambodia this proportion is estimated to be as high as 90% [35]. In Tanzania and Burkina Faso, a clear majority of the population obtains medicines for malaria through drug shops [31,33,34]. Other conveniences, apart from accessibility, add to the attractiveness of drug shops as the first port of call. For instance, drug shops are often open at hours convenient to the community, including weekends and holidays, and in many instances they offer medicines on loan to some of their clients. The private sector, including drug shops, is, therefore, an important target for enhancing access to quality-assured ACT among different socio-economic groups.

## Enabling appropriate and rational use of ACT

To preserve the effectiveness of ACT over time, it is important that these life-saving medicines are used appropriately. A number of studies have shown that malaria case-management, particularly in the retail sector, is unsatisfactory [32,36]. The private sector, in particular drug outlets, should be supported and capacitated to provide appropriate and rational management of malaria. Integrated approaches aimed at improving understanding and treatment of malaria can lead to tangible improvements in management of malaria [37]. Building on lessons learned so far, the AMFm will work with countries and technical partners to build the skills of drug shop attendants using promising models, such as the Tanzania Accredited Drugs Dispensing Outlets (ADDOs)[36].

Regulation can play an important role in enhancing access to ACT and improving the quality of care. A number of studies have reported that subsidizing ACT may need to be supported by effective regulatory policies for the intervention to be effective in crowding-out less effective anti-malarials from the market [35,38]. Countries in AMFm Phase 1 may use funds from the Global Fund to strengthen in-country regulatory systems.

Related to regulation is product quality. The AMFm will work with partners to adopt policies that assure product quality. For instance, AMFm uses the Global Fund's quality assurance policy, which requires the procurement of WHO-prequalified products and those that have passed stringent quality assessment [39].

Product branding serves to establish bonds among buyers, sellers and products. In many malaria-endemic countries there are various products available for the treatment of malaria. The availability of a wide range of products can makes it harder for buyers to distinguish quality-assured products from others. ACT under the AMFm will bear a distinct logo that will serve as an identifier and sales driver.

Finally, it is important to expand access to the parasitological confirmation of malaria, with a view to ensuring that only those who have malaria receive ACT as treatment. Most cases of presumptive treatment with ACT take place in the private sector. In the near-to medium-term, it is highly unlikely that effective public sector services will replace the private sector in most malaria-endemic countries. Therefore, universal access to diagnostics requires the achievement of universal access to these technologies in the private sector. Given the new WHO's normative guideline on the goal of universal access to diagnostics [40], it is important to identify the most suitable financing mechanisms for expanded access to diagnostics in the private sector, and to better understand the most feasible ways of expanding the use of diagnostics, particularly in the formal and informal private sectors. The operations research elements of AMFm Phase 1 provide opportunities to learn how to increase coverage of diagnostics in the private sector in a way that can inform scaling up to universal access.

## Conclusion

Access to artemisinin combination therapy remains low in most malaria endemic countries. A major impediment to access to these life-saving medicines is unaffordable prices. The Affordable Medicines Facility-malaria seeks to address the price barrier by drastically reducing the price of ACT. In addition to unaffordable prices, a variety of interrelated socio-economic factors affect prompt and effective access to ACT. The distance to facilities, locations of outlets and socio-economic status are among the factors that can hinder access to quality-assured ACT. The AMFm will support interventions to address socio-economic barriers, strengthen regulatory systems, improve supply chains and improve quality of services as a means to improve access to life-saving ACT.

## Competing interests

LM and OA are employees of the Global Fund to Fight Aids, Tuberculosis and Malaria

## Authors' contributions

Both LM and OA conceptualized this work and contributed equally to the manuscript. All authors read and approved the final manuscript.

## References

[B1] United Nations United Nations Millennium Development Goals Goal 6, Combat HIV/AIDS, malaria and other diseases http://www.un.org/millenniumgoals/aids.shtml Accessed August 8 2010

[B2] Roll Back Malaria Partnership The Global Malaria Action Plan http://www.rollbackmalaria.org/gmap/gmap.pdf Accessed August 8 2010

[B3] World Health Organization World Malaria Report 2009 http://www.who.int/malaria/world_malaria_report_2009/en/index.html Accessed August 8 2010

[B4] World Health Organization Guidelines for the treatment of malaria. Geneva 2006 12 40 http://www.who.int/malaria/publications/atoz/9789241547925/en/index.html Accessed August 8 2010

[B5] ACTwatch Group Availability, volumes, price and use of antimalarials in 7 malaria-endemic countries 2009 http://www.actwatch.info/home/home.asp Accessed August 8 2010

[B6] Arrow K Panosian C Gelband H Editors Saving Lives, Buying Time: Economics of Malaria Drugs in an Age of Resistance 2004 The National Academies Press. Washington, DC 25009879

[B7] Laxminarayan R Over M Smith D Will a global subsidy of new antimalarials delay the emergence of resistance and save lives? Health Affairs 2006 25 325 336 10.1377/hlthaff.25.2.325 16522574

[B8] The Global Fund Affordable Medicines Facility-malaria. Frequently asked questions http://www.theglobalfund.org/documents/amfm/AMFmFAQs_en.pdf Accessed August 8 2010

[B9] Management Sciences for Health (2003) Strategies for enhancing access to medicines (SEAM) http://www.msh.org/seam/5.0.htm Accessed August 8 2010

[B10] World Health Organization (2004) Equitable access to essential medicines http://whqlibdoc.who.int/hq/2004/WHO_EDM_2004.4.pdf Accessed August 8 2010

[B11] Frost L Reich MR How do good health technologies get to poor people in poor countries? 2009 Harvard University Press: Cambridge, MA

[B12] World Health Organization Global strategy and plan of action on public health, innovation, innovation and intellectual property 2008 http://apps.who.int/gb/ebwha/pdf_files/A61/A61_R21-en.pdf Accessed August 8 2010

[B13] Guyatt HL Snow RW The management of fevers in Kenyan children and adults in an area of seasonal malaria transmission Trans R Soc Trop Med Hyg 2004 98 111 115 10.1016/S0035-9203(03)00016-6 14964811

[B14] Noor AM Zurovac D Hay SI Ochola SA Snow RW Defining equity in physical access to clinical services using geographical information systems as part of malaria planning and monitoring in Kenya Trop Med Int Health 2003 8 917 926 10.1046/j.1365-3156.2003.01112.x 14516303 PMC2912492

[B15] Mbagaya GM Odhiambo MO Oniang's RK Mother's health seeking behaviour during child illness in a rural western Kenya community Afr Health Sci 2005 5 322 327 16615844 10.5555/afhs.2005.5.4.322 PMC1831955

[B16] Greenwood BM Bojang K Whitty CJ Targett GA Malaria Lancet 2005 365 1487 1498 10.1016/S0140-6736(05)66420-3 15850634

[B17] Mueller O Traore C Becher H Kouyate B Malaria morbidity, treatment-seeking behaviour, and mortality in a cohort of young children in rural Burkina Faso Trop Med Int Health 2003 8 290 296 10.1046/j.1365-3156.2003.01030.x 12667146

[B18] Snow RW Craig M Deichmann U Marsh K Estimating mortality, morbidity and disability due to malaria among Africa's non-pregnant population Bull World Health Organ 1999 77 624 640 10516785 PMC2557714

[B19] Oxford dictionaries Concise Oxford English Dictionary 11 Oxford University Press, USA Revised edition

[B20] Wasunna B Zurovac D Goodman CA Snow RW Why don't health workers prescribe ACT? A qualitative study of factors affecting the prescription of artemether-lumefantrine Malar J 2008 7 29 10.1186/1475-2875-7-29 18252000 PMC2266770

[B21] Bryce J Boschi-Pinto C Shibuya K Black RE WHO estimates of the causes of death in children Lancet 2005 365 1147 1152 10.1016/S0140-6736(05)71877-8 15794969

[B22] Larson BA Amin AA Noor AM Zurovac D Snow RW The cost of uncomplicated childhood fevers to Kenyan households: implications for reaching international access targets BMC Public Health 2006 6 314 10.1186/1471-2458-6-314 17196105 PMC1770919

[B23] Worrall E Basu S Hanson K Is malaria a disease of poverty? A review of the literature Trop Med Int Health 2005 10 1047 1059 10.1111/j.1365-3156.2005.01476.x 16185240

[B24] Unger JP d'Alessandro U De Paepe P Green A Can malaria be controlled where basic health services are not used? Trop Med Int Health 2006 11 314 322 10.1111/j.1365-3156.2006.01576.x 16553911

[B25] Mueller DH Abeku TA Okia M Rapuoda R Cox J Costs of early detection systems for epidemic malaria in highland areas of Kenya and Uganda Malar J 2009 8 17 10.1186/1475-2875-8-17 19149878 PMC2636824

[B26] Whitty CJ Chandler C Ansah E Leslie T Staedke SG Deployment of ACT antimalarials for treatment of malaria: challenges and opportunities Malar J 2008 7 Suppl 1 S7 10.1186/1475-2875-7-S1-S7 19091041 PMC2604871

[B27] Chibwana AI Mathanga DP Chinkhumba J Campbell CH Socio-cultural predictors of health-seeking behaviour for febrile under-five children in Mwanza-Neno district, Malawi Malar J 2009 8 219 10.1186/1475-2875-8-219 19778433 PMC2763003

[B28] Dillip A Hetzel MW Gosoniu D Kessy F Lengeler C Mayumana I Mshana C Mshinda H Schulze A Makemba A Pfeiffer C Weiss MG Obrist B Socio-cultural factors explaining timely and appropriate use of health facilities for *degedege *in south-eastern Tanzania Malar J 2009 8 144 10.1186/1475-2875-8-144 19563640 PMC2712476

[B29] Hetzel MW Obrist B Lengeler C Msechu JJ Nathan R Dillip A Makemba AM Mshana C Schulze A Mshinda H Obstacles to prompt and effective malaria treatment lead to low community-coverage in two rural districts of Tanzania BMC Public Health 2008 8 317 10.1186/1471-2458-8-317 18793448 PMC2564938

[B30] Chuma J Abuya T Memusi D Juma E Willis Akhwale W Ntwiga J Nyandigisi A Tetteh G Shretta R Amin A Reviewing the literature on access to prompt and effective malaria treatment in Kenya: implications for meeting the Abuja targets Malar J 2009 8 243 10.1186/1475-2875-8-243 19863788 PMC2773788

[B31] Goodman C Kachur SP Abdulla S Mwageni E Nyoni J Schellenberg JA Mills A Bloland P Retail supply of malaria-related drugs in rural Tanzania: risks and opportunities Trop Med Int Health 2004 9 655 63 10.1111/j.1365-3156.2004.01245.x 15189455

[B32] Maslove DM Mnyusiwalla A Mills EJ McGowan J Attaran A Wilson K Barriers to the effective treatment and prevention of malaria in Africa: a systematic review of qualitative studies BMC Int Health Hum Rights 2009 9 26 10.1186/1472-698X-9-26 19852857 PMC2782321

[B33] Tipke M Diallo S Coulibaly B Störzinger D Hoppe-Tichy T Sie A Müller O Substandard anti-malarial drugs in Burkina Faso Malar J 2008 7 95 10.1186/1475-2875-7-95 18505584 PMC2426704

[B34] World Health Organization The World Malaria Report 2008 http://www.who.int/malaria/publications/atoz/9789241563697/en/index.html Accessed August 8 2010

[B35] Yeung S Van Damme W Socheat D White N Mills A Access to artemisinin combination therapy for malaria in remote areas of Cambodia Malar J 2008 7 96 10.1186/1475-2875-7-96 18510724 PMC2430580

[B36] Rutta E Senauer K Johnson K Adeya G Mbwasi R Liana J Kimatta S Sigonda M Alphonce E Creating a new class of pharmaceutical services provider for underserved areas: the Tanzania accredited drug dispensing outlet experience Prog Community Health Partnersh 2009 3 145 153 10.1353/cpr.0.0063 20208262

[B37] Alba S Hetzel MW Goodman C Dillip A Liana J Mshinda H Lengeler C Improvements in access to malaria treatment in Tanzania after switch to artemisinin combination therapy and the introduction of accredited drug dispensing outlets - a provider perspective Malar J 2010 9 164 10.1186/1475-2875-9-164 20550654 PMC2910018

[B38] Sabot O Yeung S Pagnoni F Gordon M Petty N Schmits K Talisuna A Distribution of artemisinin-based combination therapies through private sector channels: Lessons from four country case studies http://www.rff.org/RFF/Documents/RFF-DP-08-43_FINAL.pdf Accessed October 4 2010

[B39] The Global Fund Quality Assurance of Pharmaceutical Products http://www.theglobalfund.org/en/procurement/pharmaceutical/?lang=en Accessed August 8, 2010

[B40] World Health Organization WHO guidelines for treatment of malaria 2006 http://www.who.int/malaria/docs/TreatmentGuidelines2006.pdf Accessed August 8 2010

